# Structure-Functional Study of Tyrosine and Methionine Dipeptides: An Approach to Antioxidant Activity Prediction

**DOI:** 10.3390/ijms161025353

**Published:** 2015-10-23

**Authors:** Anna Torkova, Olga Koroleva, Ekaterina Khrameeva, Tatyana Fedorova, Mikhail Tsentalovich

**Affiliations:** 1 A. N. Bach Institute of Biochemistry of the Russian Academy of Sciences, Leninsky Prospekt, 33, bld 2, Moscow 119071, Russian; E-Mails: anna_torkova@mail.ru (A.T.); fedorova_tv@mail.ru (T.F.); snowsurfers1@gmail.com (M.T.); 2 Department of Bioengineering and Bioinformatics, Moscow State University, GSP-1, Leninskie Hills, bld 73, Moscow 119234, Russian; E-Mail: khrameeva@genebee.msu.ru

**Keywords:** antioxidant capacity, methionine dipeptides, tyrosine dipeptides, antioxidant descriptors, quantum-chemical calculations

## Abstract

Quantum chemical methods allow screening and prediction of peptide antioxidant activity on the basis of known experimental data. It can be used to design the selective proteolysis of protein sources in order to obtain products with antioxidant activity. Molecular geometry and electronic descriptors of redox-active amino acids, as well as tyrosine and methionine-containing dipeptides, were studied by Density Functional Theory method. The calculated data was used to reveal several descriptors responsible for the antioxidant capacities of the model compounds based on their experimentally obtained antioxidant capacities against ABTS (2,2′-Azino-bis-(3-ethyl-benzothiazoline-6-sulfonate)) and peroxyl radical. A formula to predict antioxidant activity of peptides was proposed.

## 1. Introduction

Oxidative stress plays an important role in the aging processes of living organisms [1,2,3]. It is associated with excessive production of reactive oxygen species (ROS), which damage biomolecules (lipids, proteins, nucleic acids). The main role in the protection of these biomolecules belongs to enzymes and low-molecular weight endogenous and exogenous antioxidants [4]. Natural antioxidants (carotenoids, thiol, phenolic compounds and peptides) are used in the food industry, enriching food with antioxidant properties without toxic or cumulative effects.

Experimental study of the antioxidant properties of all possible peptides does not appear feasible [5], however quantum chemical methods allow screening and prediction of peptide antioxidant activity on the basis of known experimental data. Identification of structural and energy descriptors that define the activity of known antioxidant peptides allows the identification of new antioxidant peptides in protein sequences.

One of the central problems in the study of antioxidants is to establish structure-functional patterns and possible mechanisms of antioxidant action based on the known experimental data. In the present paper, the objects of study were free amino acids, acetate and amide derivatives of amino acids modeling the peptide bonds, and dipeptides of methionine and tyrosine. Structure-functional characterization of the antioxidant properties of dipeptides was based on experimental antioxidant capacities of the model substances, as well as their electronic and molecular descriptors defined using the quantum-chemical calculations.

To develop a rational strategy for searching the promising peptide antioxidants it is necessary to investigate how they interact with reactive oxygen species and model free radicals. Despite the structural differences of antioxidants, they basically interact with ROS via hydrogen atom transfer mechanism or single electron transfer mechanism (HAT or SET), though for most of them the antioxidant activity is implemented by a more complex mechanism: sequential proton loss electron transfer and single electron transfer followed by proton transfer (SPLET and SET-PT) [6,7,8]. The registered antioxidant effect is often affected by the intermediates interacting with each other or with free radicals, which creates a complexity in the analysis of experimental data.

Peptide antioxidant descriptors known to date include:

• The presence of redox-active amino acids (Tyr, Trp, Met, Cys, His) [9,10].
• The polar surrounding of Met [11].
• A limit on the length of the amino acid chain (the most active peptides are 3–10 amino acids long) [9,12,13].
• In the system with linoleic acid auto-oxidation—the presence of hydrophobic amino acid residues (Val, Leu, Ile) at C-terminal—as well as the presence of His, Asp, Glu, Lys, Arg [14,15,16].

It should be noted that for each experimental model, the set of structural descriptors of peptide antioxidant properties can vary significantly, since the amount of registered antioxidant effect is defined as the reactivity of used ROS or model synthetic radicals, and depends on the registration method. Therefore, when selecting the structural descriptors that determine the peptide antioxidant properties, a prerequisite is to use them only in the context of the given experimental model. Also, the positional effects and the effects of mutual influence of amino acid residues on the antioxidant properties of the peptides are currently poorly understood.

The experimental data on antioxidant properties of peptides is basically obtained *in vitro*. To date, more than 40 *in vitro* methods are known to assess the antioxidant capacity of individual compounds and mixtures [17,18,19,20]. ABTS (2,2′-Azino-bis-(3-ethyl-benzothiazoline-6-sulfonate)) radical cation and peroxyl radical are widely used *in vitro* to characterize natural antioxidants, the corresponding assays known as TEAC (Trolox Equivalent Antioxidant Capacity) and ORAC (Oxygen Radical Absorbance Capacity).

The aim of the present work is structural-functional analysis and investigation of the antioxidant action mechanisms of peptides in systems with different types of radicals:

(1) Investigation of the antioxidant activity of redox-active amino acids and their derivatives, tyrosine and methionine-containing dipeptides against ABTS cation radicals and peroxyl radicals;

(2) The study of electronic and molecular descriptors of redox-active amino acids, tyrosine and methionine-containing dipeptides by Density Functional Theory method.

## 2. Results and Discussion

One of the central problems in the study of antioxidants is to establish structure-functional patterns and possible mechanisms of antioxidant action based on the known experimental data. In the present paper, the objects of study were free amino acids, acetate and amide derivatives of amino acids modeling the peptide bonds, and dipeptides of methionine and tyrosine. Structure-functional characterization of the antioxidant properties of dipeptides was based on experimental antioxidant capacity (AOC) data of the model substances, as well as quantum-chemical calculations of the molecular and electronic descriptors. The selection of the analyzed descriptors was based on the existing data on electronic and thermodynamic parameters of the process of electrophilic substitution in aromatic systems (Mulliken charge distribution, electronegativity χ, and electrophilicity ω), as well as mechanisms of hydrogen atom transfer (bond dissociation energy, BDE), and single electron transfer (ionization potential, IP).

### 2.1. Antioxidant Properties of Amino Acids and Their Derivatives

The results of the AOC analysis against peroxyl radical (ORAC assay) and ABTS cation-radical (TEAC assay) for free amino acids are presented in Table 1. The antioxidant activity of individual amino acids is largely determined by the type of radical and conditions of analysis. In the ORAC assay the AOC decreased in a row Trp > Tyr > Met = Cys > His. Other amino acids in concentrations <1 mm did not show antioxidant properties against the peroxyl radical; this is consistent with data in the literature [21] (Trp > Met > Cys) and [22] (Trp > Tyr > Cys > Met > His). The AOC of amino acids and peptides significantly depends on the conditions of the analysis, in particular, on the ratio between the initial concentrations of azo-initiator AAPH and fluorescein in the reaction environment, which creates additional difficulties in comparing the data from different sources [23,24,25].

**Table 1:** AOC of free amino acids against peroxyl radical (ORAC) and ABTS cation radical (ТEАC).

Amino Acid	AOC, µmol TE/µmol	
	ORAC (M ± SD)	TEAC (M ± SD)
Tyr	1.02 ± 0.03	3.38 ± 0.07
Trp	2.79 ± 0.05	3.33 ± 0.05
Cys	0.49 ± 0.04	2.04 ± 0.12
Met	0.49 ± 0.03	0
His	0.078 ± 0.003	0

AOC: antioxidant capacity; ORAC: oxygen radical absorbance capacity; TEAC: trolox equivalent antioxidant capacity.

Only Cys, Tyr and Trp exhibited antioxidant activity in TEAC assay in concentrations <1 mM (Table 1), due to the lower reactivity of the ABTS cation-radical (AFP 0.68) compared with the peroxyl radical (ORP 1.00) [26,27]. AOC decreased in the order Trp ≈ Tyr > Cys, which fully agrees with the data of Clausen *et al.* [22]. Methionine did not possess antioxidant properties with respect to the cation-radical ABTS, which is fully consistent with data in the literature [28,29]. The obtained AOC values for Trp, Tyr and Cys in TEAC assay are 2.0, 5.0 and 5.0 times higher than those previously reported [29,30,31], and were associated with an increased reaction time: 40 min instead of 5–6 min.

**Figure 1:**
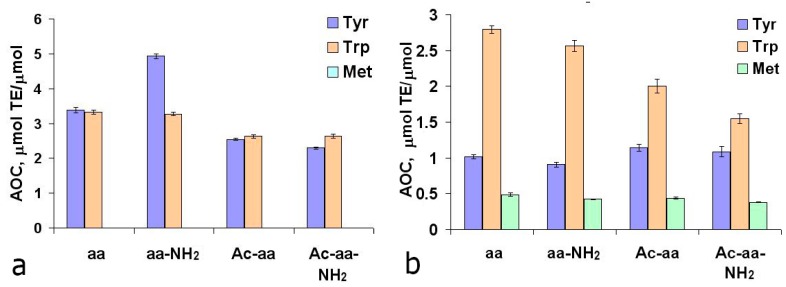
Antioxidant capacity of acetylated (Ac) and/or amidated (NH_2_) amino acids in TEAC (**a**) and ORAC (**b**) assays.

Analysis of the antioxidant properties of the peptide bond models—acetylated and/or amidated amino acids—showed the following pattern (Figure 1):

• acetylation of Tyr and Trp reduces AOC against ABTS^∙+^ 1.13 times;
• amidation of Tyr increases AOC against ABTS^∙+^ 1.4 times;
• Any modifications of Trp decreases AOC against ROH;
• Zero AOC against ABTS^∙+^ was observed for Met and its derivatives.

Among the redox-active amino acids, Tyr and Met were chosen for further research because of the dissimilar tendencies they demonstrate in TEAC and ORAC assays: while Tyr exhibited the highest AOC against peroxyl radical and low AOC against ABTS, Met possessed no activity in the ORAC assay and was rather active against ABTS.

### 2.2. Antioxidant Properties of Tyrosine Dipeptides

**Figure 2:**
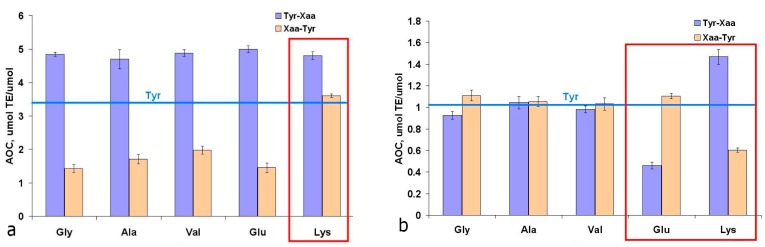
Antioxidant capacity of tyrosine dipeptides in TEAC (**a**) and ORAC (**b**) assays. The red squares indicate peculiar behavior of dipeptides with ionogenic side chains.

To confirm the obtained data (Figure 1) on the influence of tyrosine C- and N-terminal modifications on its AOC, we investigated the antioxidant properties of tyrosine dipeptides with aliphatic amino acids. In the TEAC assay, the average AOC values for dipeptides with N- and C-terminal tyrosine were 4.81 ± 0.10 and 1.70 ± 0.27 µmol TE/µmol, respectively. Dipeptides containing the N-terminal tyrosine residue exhibited, on the average, 1.4 times higher AOC values than the free tyrosine (horizontal line in Figure 2). This testifies to the important role of the-amino group, the presence of which, apparently, determines the mechanism of interaction of tyrosine residues with cation-radical ABTS and the set of resulting products. Dipeptides with C-terminal Tyr exhibit half the AOC values compared with free tyrosine (Figure 2a). Data on the influence of the position of Tyr on the AOC of dipeptides resembles the data on the photo-oxidation of tyrosine peptides: the rate constant for the photooxidation of Tyr-Gly is 1.1 times higher than that of Tyr, and for Gly-Tyr—1.7 times lower at pH = 10.0 [32].

Lys-Tyr exhibited twice the AOC value compared with other dipeptides with C-terminal Tyr (Figure 2a), which was apparently caused by the interaction of phenolic hydroxyl in Tyr with ε-aminogroup of Lys. However, another dipeptide containing an ionogenic side chain (Glu-Tyr) had similar AOC with the rest of dipeptides with C-terminal Tyr, indicating no interaction between the phenolic hydroxyl Tyr with a carboxyl group of Glu (Figure 2a).

In ORAC assay, the AOC values of tyrosine dipeptides with aliphatic amino acids was practically no different from free tyrosine AOC, and was around 1 µmol TE/µmol. However, for dipeptides with ionogenic groups in the side radicals (Glu, Lys) we observed a significant influence of the relative positions of amino acid residues (Figure 2b).

**Figure 3:**
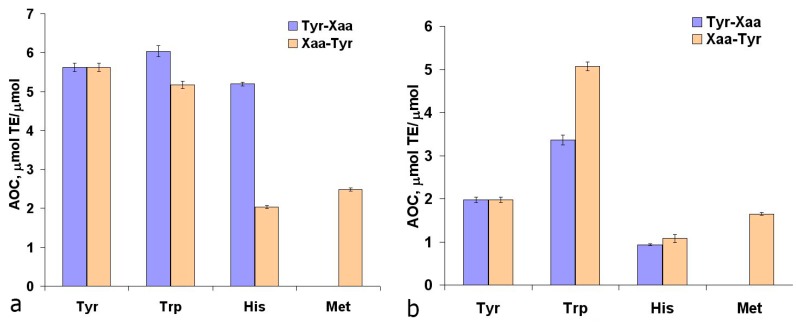
Mutual effects of redox-active amino acid residues on the antioxidant capacity of tyrosine dipeptides against the ABTS cation-radical (**a**) and the peroxyl radical (**b**).

Also, the AOC analysis of Tyr dipeptides with the residues of redox-active amino acids Trp, Met, and His was conducted (Figure 3). Based on the AOC values of N- and C-terminal Trp, Tyr, Met, and His residues (Figure 1a and Figure 2a), the theoretical AOC values of the corresponding tyrosine dipeptides were calculated for the TEAC assay. These theoretical AOC values were compared to the experimental data to reveal additive effect for Trp-Tyr (5.0 *vs*. 5.17 µmol TE/µmol) and infra-additive effects for Tyr-Tyr (6.48 *vs*. 5.62 µmol TE/µmol) and Tyr-Trp (7.41 *vs*. 6.04 µmol TE/µmol). The reduced AOC of the Tyr-Tyr and Tyr-Trp dipeptides compared with the combined AOC of the comprising amino acids appears to result from the interactions of the redox-active amino acid residues with each other. Also for Met-Tyr, Tyr-His and His-Tyr a synergic effect was revealed (Figure 3a), caused, apparently, by the processes of intramolecular tunneling electron transfer from methionine or histidine residues to the Tyr phenoxyl radical, which was earlier observed by different authors for His-, Met-, and Tyr-containing peptides [33,34,35,36,37,38].

In the case of the ORAC assay, the additive AOC effect was revealed for all dipeptides with an exception of Trp-Tyr, where a synergic effect was indicated (3.6 *vs*. 5.1 µmol TE/µmol). The effect of intramolecular electron transfer in Trp-Tyr from Tyr residue to the tryptophan radical was indicated earlier [39]. In the photooxidation of Trp-Tyr in a saturated N_2_O solution, after 0.1 ms after the laser pulse, the absorption spectrum recorded a peak at 530 nm, corresponding to the Trp radical, with a subsequent decrease in optical density in this area and the increase in optical density at 410 and 300 nm, which indicates the formation of Tyr radical [40].

Based on these data it was suggested that the effect of intramolecular synergism in the interaction of Trp-Tyr dipeptide with peroxyl radical is due to intramolecular electron transfer from the phenolic hydroxyl of tyrosine to Trp radical. The said intramolecular electron transfer also competes with the direct oxidation of Tyr residue by peroxyl radical. The data obtained explains the results of Hernandez-Ledesma *et al.* [21,23], who showed that the structural fragments of antioxidant peptides from protein whey (WY, WYS, WYSLA and others) have higher AOE values against the peroxyl radical compared to equimolecular mixture of amino acids that make up their composition.

Summarizing the results of this section, it should be noted that the structural descriptors that define high AOC of the tyrosine peptides differ for the two assays, TEAC and ORAC. In the case of cation-radical ABTS, the dipeptide must not contain Met for N-terminal Tyr, and Lys/Trp for C-terminal Tyr. In the case of peroxyl radical, a dipeptide with high AOC must contain Lys/Arg for the N-terminal Tyr, and Trp for the C-terminal Tyr.

### 2.3. Antioxidant Properties of Methionine Dipeptides

**Figure 4:**
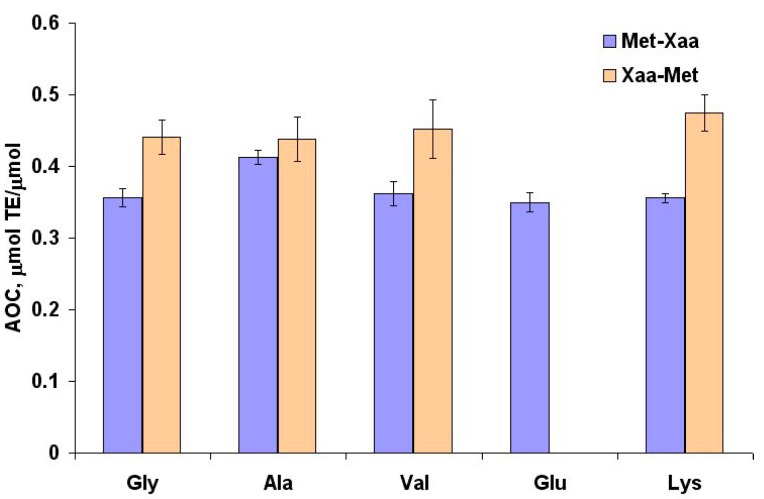
Antioxidant capacity of methionine dipeptides against peroxyl radical.

AOC values against peroxyl radical for methionine dipeptides with aliphatic and ionogenic amino acids are shown on Figure 4. The dipeptides with C-terminal Met show AOC similar to that of free Met (0.45 µmol TE/µmol *vs*. 0.49 ± 0.03 µmol TE/µmol), whereas dipeptides with N-terminal Met showed 20% lower AOC, which indicates involvement of the methionine carboxyl group in interaction with peroxyl radical.

**Figure 5:**
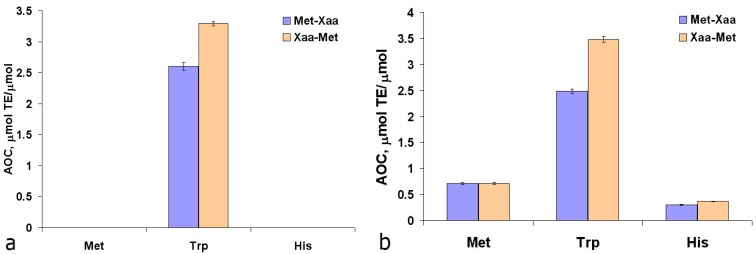
Mutual effects of redox-active amino acid residues on the antioxidant capacity of methionine dipeptides against the ABTS cation-radical (**a**) and the peroxyl radical (**b**).

In dipeptides formed by two redox-active amino acids the AOC values against peroxyl radical were additive for Met-Trp and Trp-Met, and infra-additive for Met-Met (Figure 5).

The AOC values of Met-Trp (2.60 μmol TE/µmol) and Trp-Met (3.30 µmol TE/µmol) against the ABTS cation-radical (Figure 5a) are similar to the AOC values of N- and C-terminal Trp residues (Figure 1a), indicating that Met residues are not involved in the interaction of these dipeptides with the ABTS cation-radical. Thus, the descriptor defining the high AOC of methionine peptides against the peroxyl radical is the C-terminal Met position.

### 2.4. Calculations of Molecular and Electronic Descriptors for Redox-Active Amino Acids

**Figure 6:**
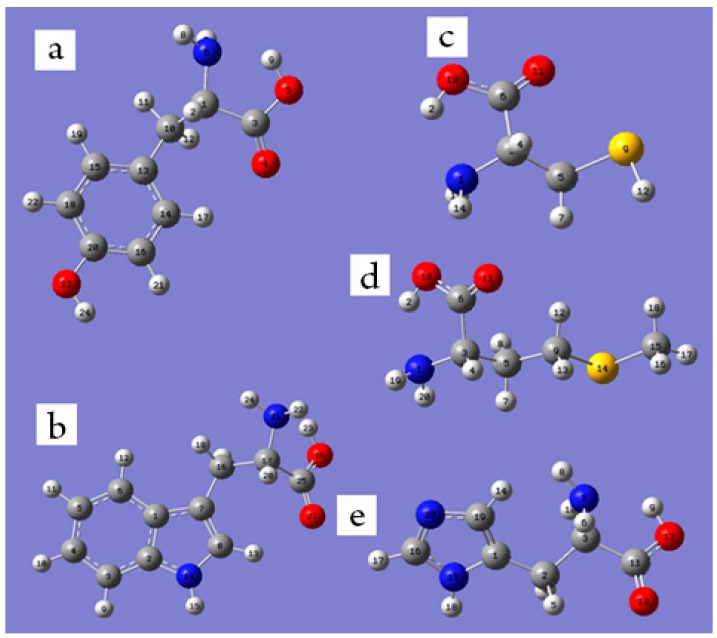
Three-dimensional structures of the redox-active l-amino acids in the gas phase-Tyr (**a**); Trp (**b**); Cys (**c**); Met (**d**); His (**e**). The C atoms are shown as grey, H—white, O—red, N—blue, S—yellow.

The gas phase three-dimensional structures of the redox-active amino acids are presented in Figure 6. The structures of all redox-active amino acids are stabilized by a hydrogen bond between carboxyl and amino group.

Table 2, Table 3, Table 4, Table 5 and Table 7 contain the data on Mulliken charge distribution for Cys, Trp, Tyr, His, Met and the corresponding radicals and cation-radicals. The comparison to the literature data [41] has revealed the same structures for Tyr and Met, and the Mulliken charge values are also in overall accordance with [41] for Met: the Mulliken charges at C9, C5, and S were, correspondingly, 0.014 (0.51), −0.224 (−0.292), and −0.15 (−0.186). Unfortunately, the authors of [41] did not calculate the Mulliken charge distribution in the benzene ring of Tyr, thus we cannot compare our results for Tyr to theirs. The analysis of Table 2, Table 3, Table 4, Table 5, Table 6 and Table 7 revealed the following features:

Cation-radicals Cys and Met are characterized by a lower electron density on the S atom compared to the uncharged molecules (Table 2 and Table 7).

The largest positive Mulliken charge on the sulfur atom in the Cys cation radical is 3.3 times higher in comparison with the uncharged radical, which indicates that the Cys cation radical is unstable and easily deprotonates (Table 7).

**Table 2:** The Mulliken charge distribution in Met and corresponding radical and cation radical.

Ionization State	Mulliken Charge Value			
	S14 *	C9	C5	C3
Met	0.014	−0.224	−0.150	−0.351
Met cation radical	0.260	−0.289	−0.444	−0.279

* Note: the atom numbering is according to Figure 6d.

**Table 3:** The Mulliken charge distribution in Tyr and corresponding radical and cation radical.

Atom	Mulliken Charge Value		
	Tyr	Tyr Radical	Tyr Cation Radical
C13 *	−0.010	−0.028	−0.006
C14	−0.104	−0.066	−0.058
C16	−0.177	−0.175	−0.122
C20	0.257	0.211	0.285
C18	−0.165	−0.170	−0.130
C15	−0.139	−0.102	−0.030
О23	−0.622	−0.362	−0.542
Н24	0.370	-	0.416

* Note: the atom numbering is according to Figure 6a.

**Table 4:** The Mulliken charge distribution in Trp and corresponding radical and cation radical.

Atom	Mulliken Charge Value		
	Trp	Trp Radical	Trp Cation Radical
C1 *	1.342	0.607	0.653
C2	−0.419	−0.580	−0.512
C3	−0.687	−0.392	−0.385
C4	−0.143	−0.218	0.004
C5	−0.548	−0.373	−0.384
C6	−0.638	−0.504	−0.381
C7	1.225	0.995	0.682
C8	−0.431	0.026	−0.075
N14	−0.052	−0.008	−0.191
H15	0.292	-	0.369

* Note: the atom numbering is according to Figure 6b.

**Table 5:** The Mulliken charge distribution in His and corresponding radical and cation radical.

Ionization State	Mulliken Charge Value				
	C1 *	C19	C16	N20	N15
His	0.775	−0.533	0.153	−0.134	−0.173
His radical	0.923	−0.324	0.141	−0.096	−0.154
His cation radical	0.451	0.164	0.221	−0.135	−0.272

* Note: the atom numbering is according to Figure 6e.

**Table 6:** Thermodynamic and energy parameters of redox-active amino acids in gas phase (298 K).

Parameter	Value				
	Tyr	Trp	Cys	Met	His
BDE, kcal/mol	83.50	90.39	106.99	-	89.82
IPe, eV	7.77	7.15	8.67	8.43	8.21
IPo, eV	6.55	5.80	6.76	6.22	6.67
E_HOMO_, eV	−6.55	−5.80	−6.76	−6.22	−6.67
E_LUMO_, eV	−0.971	−0.577	−1.102	−0.776	−0.894
χ, eV	3.762	3.186	3.933	3.496	3.783
η, eV	2.791	2.609	2.830	2.721	2.889
ω, eV	0.634	0.486	0.683	0.562	0.619
(E_HOMO_–E_LUMO_), eV	5.58	5.22	5.66	5.44	5.79

**Table 7:** The Mulliken charge distribution in Cys and corresponding radical and cation radical.

Ionization State	Mulliken Charge Value			
	S9 *	C5	C3	H12
Cys	−0.035	−0.526	0.174	0.028
Cys radical	0.093	−0.650	−0.175	-
Cys cation radical	0.313	−0.472	−0.207	0.145

* Note: the atom numbering is according to Figure 6c.

In Tyr, as well as in Tyr radical and Tyr cation radical, the maximum electron density is observed at C16 and C18 carbon atoms in *ortho*-position to the phenolic hydroxyl (Table 3), which is consistent with data in [42,43,44].

The maximum electron density in the pyrrole ring of Trp is localized on the carbon atoms C2 and C8 (Table 4). Therefore, the attack of electrophilic agents will preferably be aimed at those carbon atoms, which is consistent with the structures of the products of Trp pulse radiolysis in aqueous solutions [45].

For His, the maximum electron density is observed for the carbon atom C19, and the most pronounced decrease of the electron density upon the formation of cation-radical is also observed on C19 (Table 5). Apparently, this carbon atom is a primary target for the peroxyl radical attack.

In addition to the Mulliken charge distribution, other quantum chemical descriptors were also calculated for redox-active amino acids, including IP, BDE in proton-donor groups, energy of molecular orbitals E_HOMO_ and E_LUMO_, electronegativity χ, hardness η, and electrophylicity ω of the molecules (Table 6). Although there are a number of papers dealing with the energy descriptors of the related molecules in water [11,46,47,48], most of the published calculations were made for the gas phase [43,49,50,51], and to compare our work to the others’ results we did the same. The calculated values are in good agreement with [43,49,50,51]. Due to the structural heterogeneity of redox-active amino acids and differences in the mechanisms of their antioxidant action, significant correlations were not established between the values of their quantum-chemical descriptors (Table 6) and the AOC values in ORAC and TEAC assays (Table 1).

### 2.5. Calculations of Molecular and Electronic Descriptors for Dipeptides

For calculations of molecular and electronic descriptors of the dipeptide antioxidant properties we solved the three-dimensional structures of methionine and tyrosine-containing dipeptides in the gas phase.

**Figure 7:**
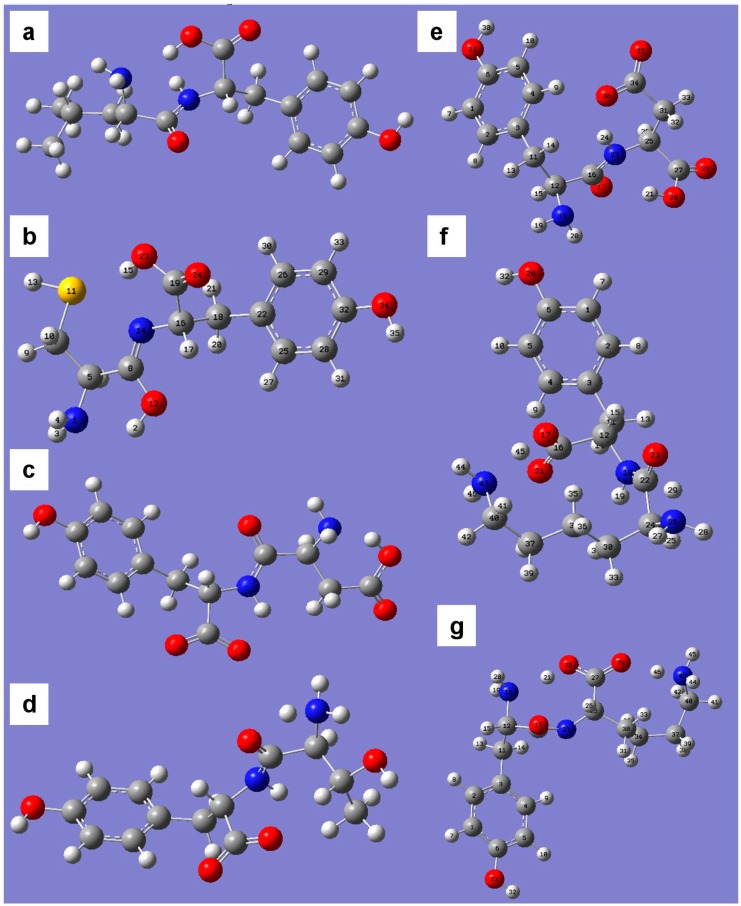
The structures of tyrosine-containing dipeptides in vacuum: Leu-Tyr (**a**); Cys-Tyr (**b**); Asp-Tyr (**c**); Thr-Tyr (**d**); Tyr-Asp (**e**); Lys-Tyr (**f**); Tyr-Lys (**g**). The C atoms are shown as grey, H—white, O—red, N—blue, S—yellow.

In the dipeptides formed by nonionic amino acids, the spatial arrangement of the side radicals makes the formation of hydrogen bonds between them impossible. The structures of dipeptides with ionogenic amino acid residues (Asp-Tyr, Tyr-Asp, Lys-Tyr, Tyr-Lys) are stabilized due to the formation of additional hydrogen bonds between atoms of the peptide bond and C-terminal carboxyl groups, guanidine and ε-amino group of Asp and Lys residues (Figure 7c,e–g).

For most of the dipeptides (Ile-Tyr, Tyr, Trp, Cys-Tyr, Val-Tyr, Tyr-His, Ser-Met, Met-Ala, Met-Gln, Met-Phe, Met-Thr, Gln-Met, Gly-Met) in the gas phase, the most thermodynamically preferred is the formation of the enol form of the peptide bond, which is stabilized with two hydrogen bonds with N- and C-terminal amino and carboxyl groups (Figure 7b). Some peptides (e.g., Thr-Tyr-, Figure 7d) form two hydrogen bonds between the atoms involved in the formation of the peptide bond and N- and C-terminal amino and carboxyl groups. In all analyzed tyrosine dipeptide structures in vacuum the spatial remoteness of phenolic hydroxyl excludes the possibility of its participation in the formation of hydrogen bonds.

#### 2.5.1. Calculations of Molecular and Electronic Descriptors for Methionine-Containing Dipeptides

**Figure 8:**
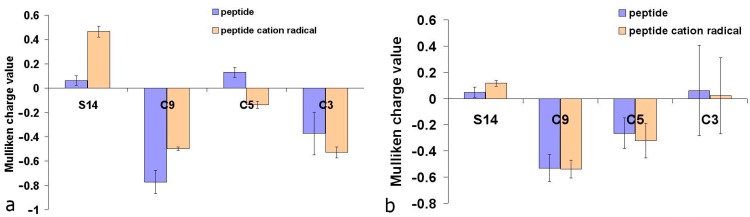
Average Mulliken charge values on sulfur and carbon atoms of methionine residues at C-terminal (**a**) and N-terminal (**b**) position in dipeptides.

The results of the calculations of the Mulliken charge distributions in the molecules of methionine-containing dipeptides in the gas phase are given in Table S1. The formation of radical cations in dipeptides with N- and C-terminal Met position leads to a 2.4 and 7.5 times increase in the positive Mulliken charge value on the sulfur atom S14 (Figure 8), together with the change of the electron density on the carbon atoms C3, C5 and C9 (numbering of atoms according to Figure 6). The maximum electron density was observed at C9. In the case of N-terminal Met, the formation of cation radical leads to an increased electron density on the C5 carbon atom and decreased electron density on C3 (Figure 8b), while Mulliken charges at C9 were similar for all ionization forms.

In C-terminal methionine dipeptides the formation of the corresponding cation radical leads to a decreased electron density on C9 and an increased electron density on C5 and C3 (Figure 8a). This indicates the unpaired electron delocalization from the sulfur atom to the atoms of the methionine carbon chain with the formation of carbon-centered radicals.

Analysis of the Mulliken charge distribution was also conducted for methionine dipeptides containing other redox-active amino acid residues: Cys, Met, His, and Trp. Compared to the uncharged Met-Met, in the Met-Met^+^ the positive Mulliken charge was significantly higher at the S atom of the C-terminal Met, which was not so for the N-terminal Met. Apparently, the C-terminal Met has a higher electron donating potential. A similar analysis was conducted for dipeptides Cys-Met, Trp-Met, His-Met. In all cases, the minimum energy belonged to those cation-radicals structures where the Met residue was electron donating: a substantial positive Mulliken charge increase on the S atom of a mercapto group was observed while the electron density distribution in Cys, Trp and His remained unchanged. The observed trend was fully confirmed by the experimental data on the antioxidant effects additivity of the redox-active amino acid residues in the Trp-Met and His-Met dipeptides in ORAC assay (Figure 5b).

For the N-terminal Met dipeptides the electron donation by Cys, Trp and His residues appears more energetically favorable. Mulliken charge on the S atom of Met was similar for the uncharged dipeptides and cation radicals, whereas electron density substantially decreased on the S atom of Cys, N20 and N15 of Hys, and N14 of Trp (the numbering of the atoms according to Figure 6).

**Figure 9:**
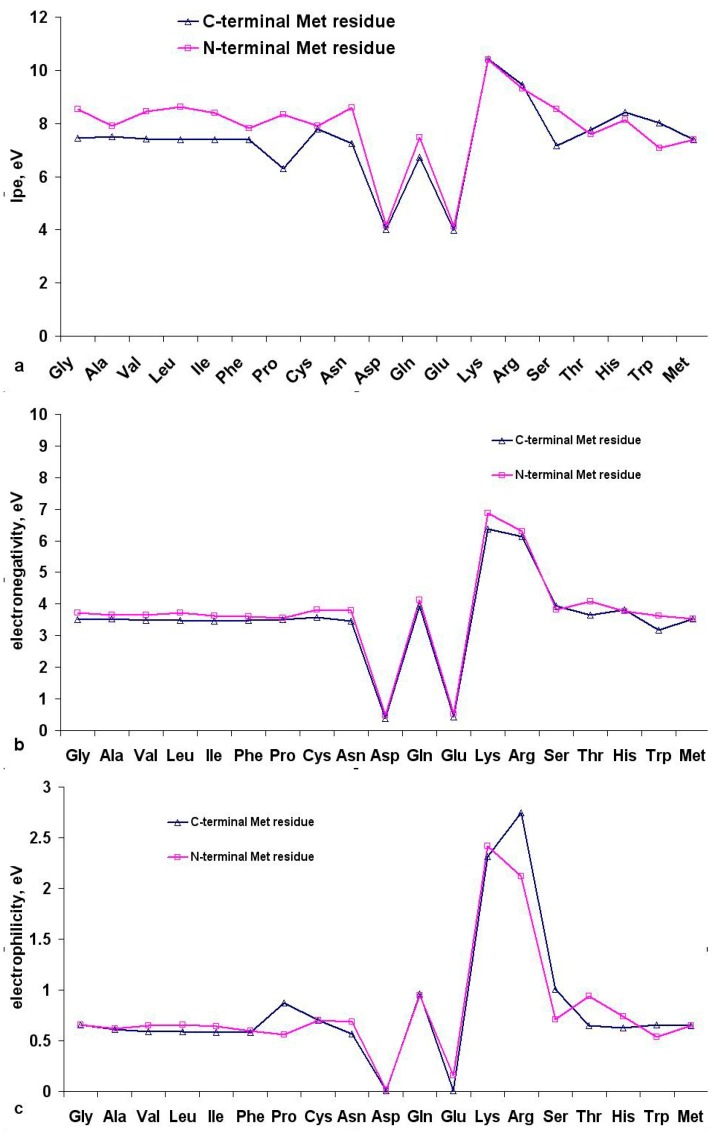
The ionization potential (**a**); electronegativity (**b**) and electrophilicity (**c**) of the dipeptides with N- and C-terminal position of methionine residues in the gas phase at 298 K.

In addition to the Mulliken charge distribution, for Met dipeptides we also calculated the parameters characterizing the stability of molecules and their electron-donating properties (Table S2, Figure 9).

Dipeptides with C-terminal Met and non-ionic residues at the N-terminal are characterized by lower values of IPe (6.35 to 7.75 eV) and electronegativity (3.50 ± 0.02 eV) compared to their analogues with N-terminal Met (IPe 7.58–8.53 eV; electronegativity χ = 3.65 ± 0.07 eV) (Figure 9a,b). Altogether these observations indicate a higher oxidation lability of dipeptides with N-terminal Met.

**Figure 10:**
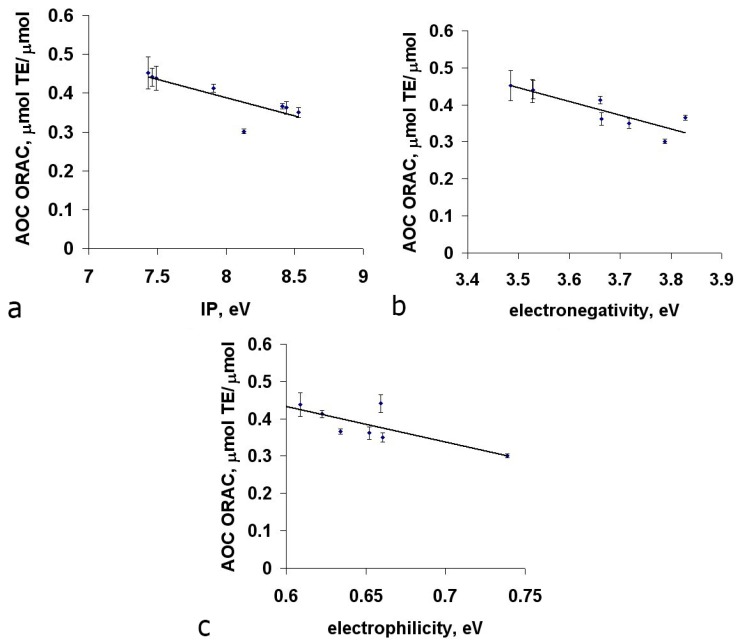
Correlation analysis between the AOC values of methionine dipeptides against peroxyl radical and the values of their ionization potentials IPe (**a**); electronegativity χ (**b**) and electrophilicity ω (**c**).

The presence of acidic residues (Glu, Asp) in the Met dipeptides leads to a two-fold reduction of the IPe, electronegativity and electrophilicity, while Lys and Arg drastically increase all three parameters, due to the corresponding negative and positive charges on ionogenic groups (Figure 9a). It should be noted that in the aquatic environment the charged groups in the side radicals of Asp, Glu, Lys and Arg are stabilized by interaction with water molecules, which leads to lower values of IP and χ in Met-Arg, Met-Lys, Lys-Met and Arg-Met dipeptides and higher values of IP and χ in Met-Glu, Met-Asp, Asp-Met and Glu-Met dipeptides. Therefore, these dipeptides were excluded from the selection for the subsequent correlation analysis between the results of quantum-chemical calculations and AOC values. For methionine dipeptides with nonionic amino acid residues we revealed the presence of an inverse correlation (significance level < 0.05) between AOC and IPe (*r* = −0.819), χ (*r* = −0.874) and ω (*r* = −0.818). The corresponding correlation fields are shown in Figure 10.

**Figure 11:**
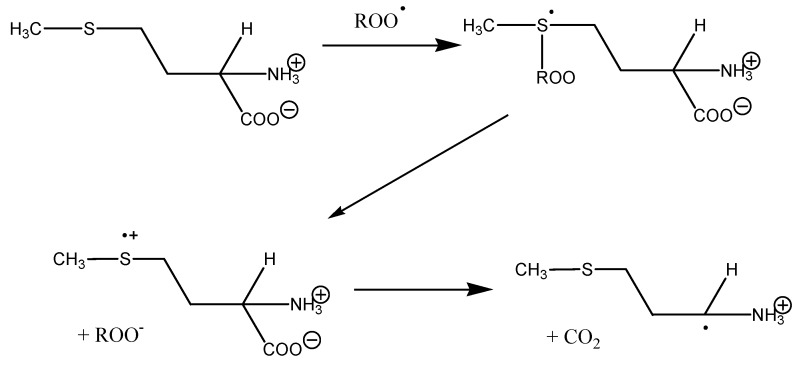
The mechanism of interaction of methionine with peroxyl radical.

In cation radicals of C-terminal Met dipeptides the highest electron density is localized at the α-carbon atom of the methionine residue. A significant inverse correlation of AOC in ORAC assay with IP value, in combination with the data on the Mulliken charge distribution in the cation radicals of methionine dipeptides, testify in favor of the mechanism of one-electron oxidation of methionine residues by peroxyl radical (Figure 11). Electron donation by the sulfur atom leads to the formation of peroxide anion and methionine cation radical. The redistribution of electron density in the radical cation of methionine leads to localization of the unpaired electron on the α-carbon atom with subsequent decarboxylation at the C-terminal position of the methionine residue. In the case of dipeptides with N-terminal Met the redistribution of electron density in the cation radical of methionine is likely to lead to peptide bond cleavage. The process of decarboxylation appears to be more energetically favorable compared with the peptide bond cleavage, which explains the higher AOC values of C-terminal Met dipeptides compared with similar N-terminal Met dipeptides (Figure 4).

#### 2.5.2. Calculations of Molecular and Electronic Descriptors for Tyrosine-Containing Dipeptides

The results of the Mulliken charge distribution calculations of the tyrosine-containing dipeptides in the gas phase are given in Table S3. The results obtained indicate the maximum electron density localized at C18 and C14 atoms of the benzene ring in N-terminal tyrosine dipeptides (Table 3). The position preferred for electrophilic attacks In C-terminal Tyr dipeptides is the C18 atom, and in the N-terminal Tyr dipeptides—both C18 and C14 atoms. Therefore, the set of resulting products can vary significantly in reactions of N- and C-terminal tyrosine dipeptides with the ABTS cation-radical, as evidenced by the differences in the experimental AOC values of the corresponding tyrosine dipeptides in TEAC assay (Figure 2a).

The Mulliken charges in the cation-radicals and radicals of tyrosine dipeptides are slightly lower on the oxygen atom of the phenolic hydroxyl compared with dipeptides, regardless of the position of Tyr residue in the dipeptide. (Table S3). Analysis of the geometry of radicals and radical cations indicates delocalization of the unpaired electron due to its π–р conjugation with the benzene ring, which naturally leads to the increased multiplicity of the C20–O23 bond and reduces its length by 1.5%–2.5%. In the radicals of tyrosine dipeptides the C20–O23 bond multiplicity was 2 (average bond length C20–O23 was 1.26 Å), and in cation-radicals 1.5 (average bond length C20–O23 was 1.34–1.36 Å).

The conjugation of unpaired electrons on the oxygen atom with the aromatic system in the radicals and cation-radicals of tyrosine-containing dipeptides leads to redistribution of Mulliken charge in the benzene ring. In the radicals of N- and C-terminal Tyr dipeptides the electron density was maximum on the C14 and C15 atoms in the meta-positions to the phenolic hydroxyl (Table 8 and Table 9), and these atoms are most preferred for the secondary attack of electrophilic agents.

**Table 8:** Carbon atoms with maximum electron density in N-terminal tyrosine dipeptides and the corresponding radicals and cation radicals.

Carbon Atom with Maximum Electron Density	Dipeptide	Phenoxyl Cation Radical of the Dipeptide	Phenoxyl Radical of the Dipeptide
C13 *	Tyr-Glu, Tyr-Arg	Tyr-Ala, Tyr-Val, Tyr-Leu, Tyr-Ile, Tyr-Phe, Tyr-Cys, Tyr-Asn, Tyr-Glu, Tyr-Ser, Tyr-Thr, Tyr-His, Tyr-Met	Tyr-Lys
C14	Tyr-Val, Tyr-Ile, Tyr-Pro, Tyr-Asn, Tyr-Asp, Tyr-Lys, Tyr-Thr, Tyr-His, Tyr-Trp	Tyr-Asp, Tyr-Gln, Tyr-Lys	Tyr-Gly, Tyr-Ala, Tyr-Met, Tyr-Val, Tyr-Leu, Tyr-Ile, Tyr-Ser, Tyr-Thr, Tyr-Pro, Tyr-Gln, Tyr-Asn, Tyr-Asp, Tyr-Phe, Tyr-Cys
C15	-	-	Tyr-Arg, Tyr-His, Tyr-Trp
C16	Tyr-Gly	Tyr-Arg	-
C18	Tyr-Tyr, Tyr-Ala, Tyr-Leu, Tyr-Phe, Tyr-Met, Tyr-Cys, Tyr-Gln, Tyr-Ser	Tyr-Gly	Tyr-Glu

* Note: carbon atom numbering according to Figure 6a.

**Table 9:** Carbon atoms with maximum electron density in C-terminal tyrosine dipeptides and the corresponding radicals and cation radicals.

Carbon Atom with Maximum Electron Density	Dipeptide	Phenoxyl Cation Radical of the Dipeptide	Phenoxyl Radical of the Dipeptide
C14 *	Arg-Tyr, Trp-Tyr	Arg-Tyr	Val-Tyr, Cys-Tyr, Asn-Tyr, Gln-Tyr, Glu-Tyr, Arg-Tyr, His-Tyr
15	-	Gly-Tyr, Ala-Tyr, Leu-Tyr, Gln-Tyr, Lys-Tyr	Ser-Tyr, Asp-Tyr, Lys-Tyr, Trp-Tyr, Met-Tyr, Thr-Tyr, Gly-Tyr, Ala-Tyr, Leu-Tyr, Ile-Tyr, Phe-Tyr, Pro-Tyr, Tyr-Tyr (C)
C18	Gly-Tyr, Ala-Tyr, Val-Tyr, Leu-Tyr, Ile-Tyr, Phe-Tyr, Pro-Tyr, Met-Tyr, Tyr-Tyr, Asn-Tyr, Asp-Tyr, Gln-Tyr, Glu-Tyr, Lys-Tyr, Ser-Tyr, Thr-Tyr, His-Tyr, Cys-Tyr	Val-Tyr, Phe-Tyr, Met-Tyr, Tyr-Tyr, Asn-Tyr, Ser-Tyr, Thr-Tyr, Pro-Tyr, Cys-Tyr, Ile-Tyr, Asp-Tyr, Glu-Tyr, His-Tyr	Tyr-Tyr (N)

* Note: carbon atom numbering according to Figure 6a.

**Figure 12:**
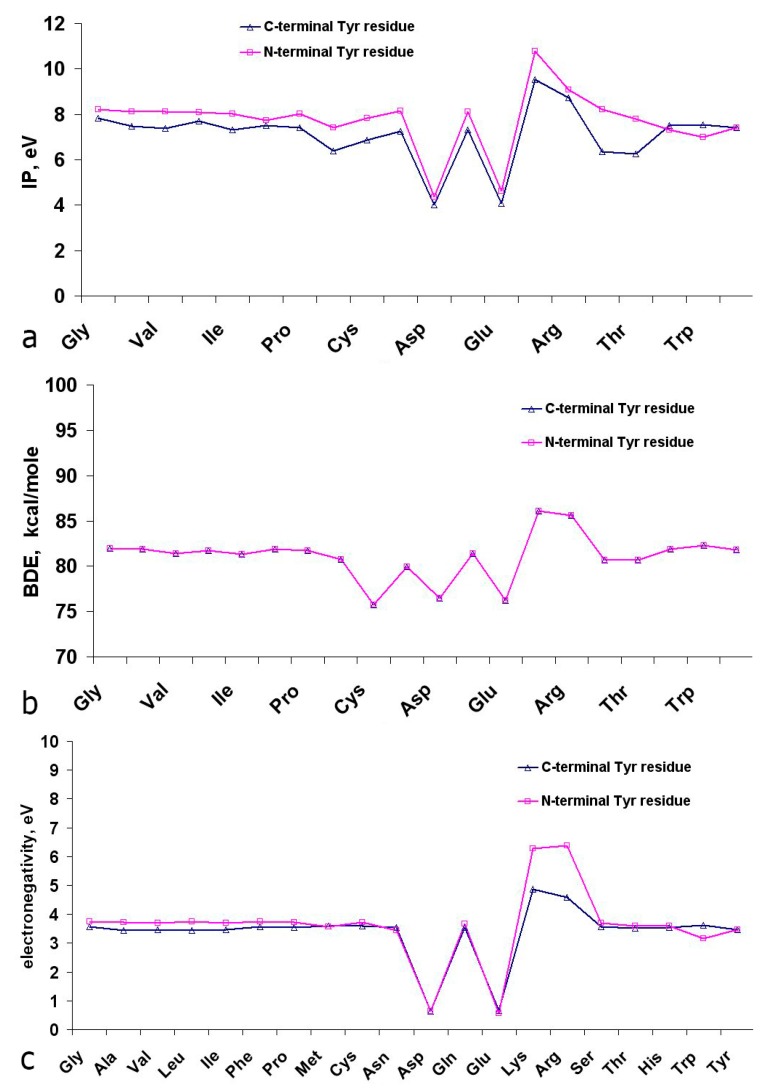
Ionization potential IPe (**a**), bond dissociation energy of phenolic O–H bond (**b**) and electronegativity (**c**) of N- and C-terminal tyrosine dipeptides in gas phase at 298 K.

The results of calculations of thermodynamic descriptors for tyrosine dipeptides with non-ionic amino acid residues in gas phase (Table S4) show that IPe (7.20 ± 0.49 eV) for C-terminal Tyr dipeptides, on average, were 10% lower compared to the N-terminal Tyr dipeptides (7.93 ± 0.38 eV) (Figure 12a). For Tyr-Glu, Tyr-Asp, Asp-Tyr and Glu-Tyr the IPe value in gas phase were lower (4.05–4.62 eV), and for Tyr-Lys, Tyr-Arg, Arg-Tyr and Lys-Tyr—higher, compared to the rest of the dipeptides (Figure 12a). A similar trend was observed for values of electronegativity (Figure 12c). The reasons for these changes in IPe and χ are described above in Section 2.5.1 during the analysis of quantum-chemical parameters of Met dipeptides.

The values of bond dissociation energy (BDE) of phenolic O–H bonds were similar for N- and C-terminal tyrosine dipeptides (83.2 ± 2.8 and 82.9 ± 5.4 kcal/mol, respectively) (Figure 12b). No significant correlations of AOC values in ORAC and TEAC assays (Figure 2 and Figure 3) *vs.* the calculated thermodynamic and energy parameters were revealed for Tyr dipeptides. This can be explained by significant differences in the structures of Tyr peptides in gas phase and aqueous solutions due to the formation of zwitterionic form of dipeptides and their interaction with the solvent molecules.

The mechanism of antioxidant action of tyrosine dipeptides is presumably similar to the proposed antioxidant mechanism for hydroxy aromatic acids. Namely, for hydroxy aromatic acids the mechanism in ORAC assay is HAT and in TEAC assay it is SPLET at pH > 4.5 and HAT at low pH [52]. Energy of the highest occupied molecular orbital, rigidity (η), and electron density on the carbon atom in metha-position to the hydroxyl are the primary descriptors of their antioxidant activity in ORAC assay, whereas in TEAC assay the primary descriptor at pH 7.4 is enthalpy of electron transfer of the phenolate ion.

The most significant descriptors of their antioxidant properties against peroxyl radical are and Mulliken charge on the carbon atom in *m*-position to the phenolic hydroxyl.

The most significant descriptor of the antioxidant properties against the ABTS radical cation at рН 7.40 is electron transfer enthalpy of the phenolate ion.

### 2.6. Search of Peptides with High AOC in Muscle/Collagen Hydrolysates

For the screening of peptides with potentially high AOC, the formula (1) was proposed to evaluate the integral parameter *I*, which determines the calculated antioxidant activity of the peptides. This parameter takes into account the detected contribution of molecular descriptors of dipeptide antioxidant properties (Table 10), the amount of redox-active amino acid residues, and the size of the peptide. It should be noted that the available literature data indicates a reduction of peptide AOC with increasing length [9,23,53,54]. The average length of antioxidant peptides described in the literature is about 10 amino acid residues.

(1) $$I=[\frac{\sum_{i=1}^{n}E_{i}}{n}]\times \frac{10}{N}$$

where *E_i_* is the value of effect according to Table 10; *n*—number of redox-active amino acid residues within peptide sequence; *N*—total amino acid length of peptide.

**Table 10:** Molecular descriptors of antioxidant activity for peptides.

Assay	Descriptor	Value of Effect
ORAC	C-terminal Met	+1
	Presence of fragments with an intramolecular synergic antioxidant effect	+1: Trp-Tyr
	Influence of ionogenic amino acids on oxred-activity of adjacent residuals	−1: Lys-Tyr, Arg-Tyr, Tyr-Glu, Tyr-Asp, +1: Tyr-Lys, Tyr-Arg
TEAC	N-terminal Tyr or Trp	+1
	Presence of fragments with an intramolecular synergic antioxidant effect	+1: Tyr-His, His-Tyr, Met-Tyr
	Influence of ionogenic amino acids on oxred-activity of adjacent residuals	+1: Lys-Tyr, Arg-Tyr

## 3. Experimental Section

### 3.1. Materials

Aminoacids, their derivatives, dipeptides, phenyl carbonic acids, solvents, and reagents were ordered from Fluka (St. Gallen, Germany) and Sigma (St. Louis, MO, USA) and Bachem (Bubendorf, Switzerland).

Inorganic salts, acids and bases of analytical grade were used. All the water solutions were prepared in de-ionized water (18.2 MΩ/cm at 25 °C, Simplicity system (Millipore, Billerica, MA, USA)).

### 3.2. Methods

#### 3.2.1. AOC Experiments

Trolox, 100 µM solution in deionized water, and 1000 µM solutions of amino acids and dipeptides in 0.01 М HCl were used.

TEAC assay was conducted according to Re *et al*. [55], by incubation of a solution of 7 mM ABTS in 2.45 mM K_2_S_2_O_6_(O_2_) in the darkness at ambient temperature for 12–18 h. The solution was further diluted with 50 mM phosphate buffered salino containing 100 mM NaCl (pH 7.40) to reach the optical density OD_734_ = 1.5 × 10^4^ L/mol/cm, which corresponds to the final concentration of ABTS ~47 µM (ε_734_ = 1.5 × 10^4^ L/mol/cm). To define AOC, 20 µM solutions of trolox or samples were added to 180 µL of ABTS^∙+^ solution. 180 µL ABTS^∙+^ solution mixed with 20 µL of deionized water was used as a control for trolox, whereas 180 µL ABTS^∙+^ solution mixed with 20 µL of 0.01 M HCl was used as a control for amino acids and peptides. The reaction was registered by the decrease of OD_734_ for 40.5 min with the measurement interval of 60 s on Synergy 2 photometer-fluorimeter (BioTek, Winooski, VT, USA).

A calibration carve for optical density aganst trolox concentration was plotted using trolox concentrations in the range 1–10 µM. The measurements were run 4 times for each concentration. Antioxidant capacity was calculated using the equation of linear regression between the concentration of trolox and the absorbance decline of ABTS^∙+^. Antioxidant capacity was quantified in μmol Trolox equivalents (TE, μM) per μM of antioxidant.

ORAC assay was conducted according to Ou *et al.* in a Moore *et al.* modification [56,57]. Peroxyl radicals were generated directly in the reaction medium during the thermal decomposition of the azo compound AAPH initiated via incubation for 10 min at 37 °C according to [58]. The reaction mixture was incubated at 37 °C for 30 s with vigorous stirring (1200 rpm) in a microtiter Thermo incubator PHMP Grant Bio (Chelmsford, UK). The fluorescence fading kinetics was registered 1h with the measurement interval of 60 s on Synergy 2 photometer-fluorimeter (BioTek, Winooski, VT, USA). Antioxidant capacity was calculated using the equation of linear regression between the concentration of trolox and net area under the curve of fluorescein decay. Antioxidant capacity was quantified in Trolox equivalents (TE, μM) per μM of antioxidant. The experiments were run in 4 replicates.

#### 3.2.2. Calculation of Molecular and Electron Descriptors by Density Functional Theory Method

The calculations were made in Gaussian 3.0 (Gaussian., Inc., Wallingford, CT, USA) [59] at the Density Functional Theory level, B3LYP hybrid functional, with 6-311++G**(d,p) basis set. Chargeless molecules, radicals, and cation radicals of amino acids and peptides were investigated. Similarly to an approach reported in [57], Mulliken charges for the key atoms, HOMO and LUMO energies (E_HOMO_ and E_LUMO_), ionization potentials (IP_O_ and IP_E_), electronegativity (χ), electrophilicity (ω) nd rigidity (η) of the species were calculated, as well as bond dissociation energy in the proton donor group (BDE).

## 4. Conclusions

It was demonstrated that the amino acids Tyr, Trp, Met, Cys and His are redox-active against peroxyl radical, and Tyr, Trp and Cys against ABTS cation radical. The presence of Tyr and Met residues in the dipeptides leads to diverse effects on their AOC values, determined by the N- or C-terminal position of the residues, their interaction with other redox-active amino acid residues and with ionic groups in the side chains. Intramolecular synergic effects were shown in interactions of Trp-Tyr with peroxyl radical, and Met-Tyr, Tyr-His, and His-Tyr with the ABTS cation radical.

Structure-functional studies showed that the primary antioxidant descriptors of tyrosine dipeptides against peroxyl radical are the HOMO energy, rigidity of the molecule, and Mulliken charge value on the carbon atom in *m*-position to the phenolic hydroxyl, and against the ABTS cation radical at pH 7.40—electron transfer enthalpy from the phenolate ion. The most important antioxidant descriptors of methionine dipeptides against peroxyl radical are the ionization potential (IP), electronegativity χ, and electrophilicity ω.

An integral parameter I was proposed to assess the antioxidant properties of peptides based on the revealed empirical descriptors.

## Supplementary Materials

Supplementary materials can be found at http://www.mdpi.com/1422-0067/16/10/25353/s1.
