# Supplementary material for: Structure-Functional Study of Tyrosine and Methionine Dipeptides: An Approach to Antioxidant Activity Prediction
Source: Int J Mol Sci. 2015 Oct 23;16(10):25353–76. doi: 10.3390/ijms161025353 (PMC4632805; doi:10.3390/ijms161025353)
Supplement: Supplementary file 1 [file ijms-16-25353-s001.pdf]

Supplementary Information

Table S1. Mulliken charge distribution in methionine-containing dipeptides and the corresponding cation radicals.

Compound	Mulliken Charge Value			
	S14	C9	C5	C3
Gly-Met z = 0	0.028	−0.609	0.198	−0.421
Gly-Met cation radical z = +1	0.480	−0.479	−0.145	−0.540
Ala-Met z = 0	0.085	−0.836	0.137	−0.299
Ala-Met cation radical z = +1	0.487	−0.480	−0.174	−0.515
Val-Met z = 0	0.091	−0.839	0.144	−0.307
Val-Met cation radical z = +1	0.481	−0.502	−0.125	−0.573
Leu-Met z = 0	0.097	−0.876	0.150	−0.297
Leu-Met cation radical z = +1	0.477	−0.483	−0.152	−0.552
Ile-Met z = 0	0.098	−0.853	0.145	−0.284
Ile-Met cation radical z = +1	0.483	−0.506	−0.117	−0.576
Phe-Met z = 0	0.092	−0.871	0.158	−0.298
Phe-Met cation radical z = +1	0.473	−0.488	−0.143	−0.549
Pro-Met z = 0	0.072	−0.835	0.149	−0.524
Pro-Met cation radical z = +1	0.455	−0.498	−0.137	−0.486
Met-Met z = 0 C/N	0.047/0.085	−0.990/−0.471	0.344/−0.443	−0.320/0.528
Met-Met cation radical z = +1 C/N	0.275/0.108	−0.669/−0.480	0.202/−0.508	−0.631/0.128
Cys-Met z = 0 Met/Cys	0.032/−0.008	−0.648/−0.511	0.325/-	−0.623/−0.235
Cys-Met cation radical z = +1 Met/Cys	0.489/0.095	−0.491/−0.430	−0.131/-	−0.532/−0.287
Asn-Met z = 0	0.094	−0.832	0.107	−0.277
Asn-Met cation radical z = +1	0.466	−0.482	−0.169	−0.586
Asp-Met z = −1	−0.002	−0.718	0.066	−0.805
Asp-Met cation radical z = 0	0.373	−0.519	−0.105	−0.426
Gln-Met z = 0	0.013	−0.741	0.272	−0.541
Gln-Met cation radical z = +1	0.481	−0.510	−0.079	−0.486
Glu-Met z = −1	0	−0.698	0.045	−0.750
Glu-Met cation radical z = 0	0.375	−0.524	−0.071	−0.442
Lys-Met z = 1	0.049	−0.625	0.092	−0.169
Lys-Met cation radical z = +2	0.529	−0.488	−0.167	−0.541
Arg-Met z = 1	0.097	−0.695	0.360	−0.850
Arg-Met cation radical z = +2	0.530	−0.488	−0.167	−0.540
Ser-Met z = 0	0.012	−0.652	0.158	−0.730
Ser-Met cation radical z = +1	0.440	−0.512	−0.120	−0.544
Thr-Met z = 0	0.095	−0.879	0.147	−0.254
Thr-Met cation radical z = +1	0.444	−0.509	−0.141	−0.508
His-Met z = 0	0.110	−0.440	−0.207	−0.334
His-Met cation radical z = +1	0.260	−0.459	−0.219	−0.304
Trp-Met z = 0	0.07	−0.410	−0.018	−0.495
Trp-Met cation radical z = +1	0.108	−0.507	0.002	−0.304
Met-Gly z = 0	−0.008	−0.459	−0.349	0.343
Met-Gly cation radical z = +1	0.117	−0.586	−0.317	0.012
Met-Ala z = 0	−0.017	−0.435	−0.361	−0.030

Table S1. *Cont.*

Compound	Mulliken Charge Value			
	S14	C9	C5	C3
Met-Ala cation radical z = +1	0.131	−0.531	−0.404	0.172
Met-Val z = 0	0.090	−0.642	−0.171	0.231
Met-Val cation radical z = +1	0.108	−0.556	−0.428	0.353
Met-Leu z = 0	0.072	−0.427	−0.446	0.440
Met-Leu cation radical z = +1	0.104	−0.519	−0.434	0.262
Met-Ile z = 0	0.083	−0.663	−0.151	0.279
Met-Ile cation radical z = +1	0.105	−0.566	−0.415	0.306
Met-Phe z = 0	0.044	−0.518	−0.223	−0.259
Met-Phe cation radical z = +1	0.114	−0.622	−0.175	−0.275
Met-Pro z = 0	0.061	−0.468	−0.269	0.156
Met-Pro cation radical z = +1	0.179	−0.536	−0.163	−0.188
Met-Cys z = 0 Met/Cys	0.086/0.021	−0.480/−0.450	−0.396/-	0.398/−0.152
Met-Cys cation radical z = +1 Met/Cys	0.116/0.326	−0.481/−0.350	−0.451/-	0.135/−0.237
Met-Asn z = 0	0.086	−0.464	−0.419	0.463
Met-Asn cation radical z = +1	0.114	−0.468	−0.533	0.200
Met-Asp z = -1	0.022	−0.319	−0.378	0.347
Met-Asp cation radical z = 0	0.089	−0.470	−0.432	0.453
Met-Gln z = 0	0.018	−0.589	−0.176	−0.369
Met-Gln cation radical z = +1	0.112	−0.621	−0.185	−0.350
Met-Glu z = −1	0.050	−0.666	−0.102	−0.277
Met-Glu cation radical z = 0	0.009	−0.591	−0.140	−0.431
Met-Lys z = 1	0.069	−0.624	−0.191	−0.281
Met-Lys cation radical z = +2	0.438	−0.436	−0.397	−0.093
Met-Arg z = 1	0.057	−0.622	−0.169	−0.229
Met-Arg cation radical z = +2	0.336	−0.455	−0.302	−0.164
Met-Ser z = 0	0.090	−0.464	−0.415	0.538
Met-Ser cation radical z = +1	0.116	−0.477	−0.461	0.160
Met-Thr z = 0	0.026	−0.585	−0.160	−0.347
Met-Thr cation radical z = +1	0.097	−0.647	−0.161	−0.316
Met-His z = 0	0	−0.452	−0.382	0.387
Met-His cation radical z = +1	−0.04	−0.517	−0.369	0.098
Met-Trp z = 0	0.033	−0.493	−0.534	0.387
Met-Trp cation radical z = +1	0.140	−0.567	−0.483	0.527

Table S2. The electron density distribution and the bond length of O–H in aromatic systems of tyrosine-containing dipeptides and related radicals and the corresponding cation-radicals.

Compound	Mulliken Charge Value			
	S14	C9	C5	C3
Gly-Met z = 0	0.028	−0.609	0.198	−0.421
Gly-Met cation radical z = +1	0.480	−0.479	−0.145	−0.540
Ala-Met z = 0	0.085	−0.836	0.137	−0.299
Ala-Met cation radical z = +1	0.487	−0.480	−0.174	−0.515
Val-Met z = 0	0.091	−0.839	0.144	−0.307
Val-Met cation radical z = +1	0.481	−0.502	−0.125	−0.573
Leu-Met z = 0	0.097	−0.876	0.150	−0.297
Leu-Met cation radical z = +1	0.477	−0.483	−0.152	−0.552
Ile-Met z = 0	0.098	−0.853	0.145	−0.284
Ile-Met cation radical z = +1	0.483	−0.506	−0.117	−0.576
Phe-Met z = 0	0.092	−0.871	0.158	−0.298
Phe-Met cation radical z = +1	0.473	−0.488	−0.143	−0.549
Pro-Met z = 0	0.072	−0.835	0.149	−0.524
Pro-Met cation radical z = +1	0.455	−0.498	−0.137	−0.486
Met-Met z = 0 C/N	0.047/0.085	−0.990/−0.471	0.344/−0.443	−0.320/0.528
Met-Met cation radical z = +1 C/N	0.275/0.108	−0.669/−0.480	0.202/−0.508	−0.631/0.128
Cys-Met z = 0 Met/Cys	0.032/−0.008	−0.648/−0.511	0.325/–	−0.623/−0.235
Cys-Met cation radical z = +1 Met/Cys	0.489/0.095	−0.491/−0.430	−0.131/-	−0.532/−0.287
Asn-Met z = 0	0.094	−0.832	0.107	−0.277
Asn-Met cation radical z = +1	0.466	−0.482	−0.169	−0.586
Asp-Met z = −1	−0.002	−0.718	0.066	−0.805
Asp-Met cation radical z = 0	0.373	−0.519	−0.105	−0.426
Gln-Met z = 0	0.013	−0.741	0.272	−0.541
Gln-Met cation radical z = +1	0.481	−0.510	−0.079	−0.486
Glu-Met z = −1	0	−0.698	0.045	−0.750
Glu-Met cation radical z = 0	0.375	−0.524	−0.071	−0.442
Lys-Met z = 1	0.049	−0.625	0.092	−0.169
Lys-Met cation radical z = +2	0.529	−0.488	−0.167	−0.541
Arg-Met z = 1	0.097	−0.695	0.360	−0.850
Arg-Met cation radical z = +2	0.530	−0.488	−0.167	−0.540
Ser-Met z = 0	0.012	−0.652	0.158	−0.730
Ser-Met cation radical z = +1	0.440	−0.512	−0.120	−0.544
Thr-Met z = 0	0.095	−0.879	0.147	−0.254
Thr-Met cation radical z = +1	0.444	−0.509	−0.141	−0.508
His-Met z = 0	0.110	−0.440	−0.207	−0.334
His-Met cation radical z = +1	0.260	−0.459	−0.219	−0.304
Trp-Met z = 0	0.07	−0.410	−0.018	−0.495
Trp-Met cation radical z = +1	0.108	−0.507	0.002	−0.304
Met-Gly z = 0	−0.008	−0.459	−0.349	0.343

Table S2. *Cont.*

Compound	Mulliken Charge Value			
	S14	C9	C5	C3
Met-Gly cation radical z = +1	0.117	−0.586	−0.317	0.012
Met-Ala z = 0	−0.017	−0.435	−0.361	−0.030
Met-Ala cation radical z = +1	0.131	−0.531	−0.404	0.172
Met-Val z = 0	0.090	−0.642	−0.171	0.231
Met-Val cation radical z = +1	0.108	−0.556	−0.428	0.353
Met-Leu z = 0	0.072	−0.427	−0.446	0.440
Met-Leu cation radical z = +1	0.104	−0.519	−0.434	0.262
Met-Ile z = 0	0.083	−0.663	−0.151	0.279
Met-Ile cation radical z = +1	0.105	−0.566	−0.415	0.306
Met-Phe z = 0	0.044	−0.518	−0.223	−0.259
Met-Phe cation radical z = +1	0.114	−0.622	−0.175	−0.275
Met-Pro z = 0	0.061	−0.468	−0.269	0.156
Met-Pro cation radical z = +1	0.179	−0.536	−0.163	−0.188
Met-Cys z = 0 Met/Cys	0.086/0.021	−0.480/−0.450	−0.396/-	0.398/−0.152
Met-Cys cation radical z = +1 Met/Cys	0.116/0.326	−0.481/−0.350	−0.451/-	0.135/−0.237
Met-Asn z = 0	0.086	−0.464	−0.419	0.463
Met-Asn cation radical z = +1	0.114	−0.468	−0.533	0.200
Met-Asp z = −1	0.022	−0.319	−0.378	0.347
Met-Asp cation radical z = 0	0.089	−0.470	−0.432	0.453
Met-Gln z = 0	0.018	−0.589	−0.176	−0.369
Met-Gln cation radical z = +1	0.112	−0.621	−0.185	−0.350
Met-Glu z = −1	0.050	−0.666	−0.102	−0.277
Met-Glu cation radical z = 0	0.009	−0.591	−0.140	−0.431
Met-Lys z = 1	0.069	−0.624	−0.191	−0.281
Met-Lys cation radical z = +2	0.438	−0.436	−0.397	−0.093
Met-Arg z = 1	0.057	−0.622	−0.169	−0.229
Met-Arg cation radical z = +2	0.336	−0.455	−0.302	−0.164
Met-Ser z = 0	0.090	−0.464	−0.415	0.538
Met-Ser cation radical z = +1	0.116	−0.477	−0.461	0.160
Met-Thr z = 0	0.026	−0.585	−0.160	−0.347
Met-Thr cation radical z = +1	0.097	−0.647	−0.161	−0.316
Met-His z = 0	0	−0.452	−0.382	0.387
Met-His cation radical z = +1	−0.04	−0.517	−0.369	0.098
Met-Trp z = 0	0.033	−0.493	−0.534	0.387
Met-Trp cation radical z = +1	0.140	−0.567	−0.483	0.527

Table S3. Thermodynamic and energy parameters of the methionine-containing dipeptides in the gas phase (298 K).

Compound	EBD, kCal/mol	IPe, eV	IPo, eV	E_{HOMO}, eV	E_{LUMO}, eV	χ, eV
Gly-Met (z = 0)	-	7.46	5.89	−5.89	−1.17	3.52
Ala-Met (z = 0)	-	7.49	6.08	−6.08	−0.97	3.53
Val-Met (z = 0)	-	7.43	6.08	−6.08	−0.89	3.48
Leu-Met (z = 0)	-	7.40	6.06	−6.06	−0.89	3.48
Ile-Met (z = 0)	-	7.40	6.07	−6.07	−0.87	3.47
Phe-Met (z = 0)	-	7.40	6.09	−6.09	−0.89	3.49
Pro-Met (z = 0)	-	6.31	5.29	−5.29	−1.73	3.51
Met-Met (z = 0)	-	7.49	5.92	−5.92	−1.15	3.53
Cys-Met (z = 0)	88.47 (Cys)	7.80	5.88	−5.88	−1.29	3.58
Asn-Met (z = 0)	-	7.26	6.09	−6.09	−0.84	3.46
Asp-Met (z = −1)	-	4.02	2.80	−2.80	2.06	0.37
Gln-Met (z = 0)	-	6.73	5.97	−5.97	−1.92	3.94
Glu-Met (z = −1)	-	3.99	2.77	−2.77	1.88	0.44
Lys-Met (z = 1)	-	10.42	8.57	−8.57	−4.18	6.38
Arg-Met (z = 1)	-	9.46	7.85	−7.85	−4.43	6.14
Ser-Met (z = 0)	-	7.15	5.85	−5.85	−2.02	3.94
Thr-Met (z = 0)	-	7.75	6.17	−6.17	−1.10	3.64
His-Met (z = 0)	98.08 (His)	8.41	6.72	−6.72	−0.94	3.83
Trp-Met (z = 0)	97.10 (Trp)	8.01	6.11	−6.11	−1.14	3.17
Met-Gly (z = 0)	-	8.53	6.33	−6.33	−1.10	3.72
Met-Ala (z = 0)	-	7.91	6.35	−6.35	−0.97	3.66
Met-Val (z = 0)	-	8.44	6.23	−6.23	−1.09	3.66
Met-Leu (z = 0)	-	8.63	6.35	−6.35	−1.11	3.73
Met-Ile (z = 0)	-	8.40	6.23	−6.23	−1.04	3.63
Met-Phe (z = 0)	-	7.81	6.33	−6.33	−0.89	3.61
Met-Pro (z = 0)	-	8.32	6.41	−6.41	−0.72	3.56
Met-Cys (z = 0)	81.44 (Cys)	7.90	6.39	−6.39	−1.23	3.81
Met-Asn (z = 0)	-	8.60	6.41	−6.41	−1.20	3.80
Met-Asp(z = −1)	-	4.15	2.56	−2.56	−1.62	0.47
Met-Gln (z = 0)	-	7.48	6.35	−6.35	−1.88	4.12
Met-Glu (z = −1)	-	4.11	2.59	−2.59	−1.54	0.53
Met-Lys (z = 1)	-	10.39	9.33	−9.33	−4.44	6.88
Met-Arg(z = 1)	-	9.30	8.66	−8.66	−3.96	6.31
Met-Ser (z = 0)	-	8.53	6.42	−6.42	−1.24	3.83
Met-Thr (z = 0)	-	7.58	6.26	−6.26	−1.87	4.07
Met-His (z = 0)	90.22 (His)	8.13	6.21	−6.21	−1.36	3.78
Met-Trp (z = 0)	86.89 (Trp)	7.08	5.50	−5.50	−0.83	3.63

Table S4. Thermodynamic and energy characteristics of the tyrosine-containing dipeptides in the gas phase (298 K).

Compound	EBD, kCal/mol	IPe, eV	IPo, eV	E_{HOMO}, eV	E_{LUMO}, eV	χ, eV
Gly-Tyr (z = 0)	81.94	7.84	6.03	−6.03	−1.11	3.57
Ala-Tyr (z = 0)	81.89	7.48	6.01	−6.01	−0.88	3.45
Val-Tyr (z = 0)	81.43	7.37	5.89	−5.89	−1.07	3.48
Leu-Tyr (z = 0)	81.74	7.69	6.00	−6.00	−0.88	3.44
Ile-Tyr (z = 0)	81.36	7.33	5.88	−5.88	−1.03	3.46
Phe-Tyr (z = 0)	81.86	7.50	6.00	−6.00	−1.11	3.56
Pro-Tyr (z = 0)	81.69	7.40	6.00	−6.00	−1.08	3.54
Met-Tyr (z = 0)	80.75	6.38	5.41	−5.41	−1.78	3.59
Cys-Tyr (z = 0) Cys/Tyr	99.6/75.78	6.86	5.89	−5.89	−1.26	3.60
Asn-Tyr (z = 0)	79.95	7.26	6.03	−6.03	−1.06	3.55
Asp-Tyr (z = −1)	76.46	4.02	2.82	−2.82	1.54	0.64
Gln-Tyr (z = 0)	81.41	7.30	5.99	−5.99	−1.11	3.55
Glu-Tyr (z = −1)	76.24	4.08	2.82	−2.82	1.49	0.67
Lys-Tyr (z = 1)	86.13	9.53	8.16	−8.16	−1.60	4.88
Arg-Tyr (z = 1)	85.60	8.73	8.01	−8.01	−1.19	4.60
Ser-Tyr (z = 0)	80.65	6.36	5.28	−5.28	−1.88	3.58
Thr-Tyr (z = 0)	80.66	6.27	5.27	−5.27	−1.78	3.52
His-Tyr (z = 0) His/Tyr	-/81.89	7.51	6.02	−6.02	−1.07	3.55
Trp-Tyr (z = 0) Trp/Tyr	84.20/82.30	7.55	6.19	−6.19	−1.08	3.63
Tyr-Tyr (z = 0) N-Tyr/C-Tyr	89.21/81.79	7.41	6.00	−6.00	−0.92	3.46
Tyr-Gly (z = 0)	83.66	8.21	6.59	−6.59	−0.93	3.76
Tyr-Ala (z = 0)	83.63	8.13	6.56	−6.56	−0.90	3.73
Tyr-Val (z = 0)	80.69	8.11	6.53	−6.53	−0.88	3.70
Tyr-Leu (z = 0)	81.00	8.10	6.58	−6.58	−0.91	3.75
Tyr-Ile (z = 0)	80.64	8.03	6.54	−6.54	−0.88	3.71
Tyr-Phe (z = 0)	89.00	7.72	6.57	−6.57	−0.93	3.75
Tyr-Pro (z = 0)	83.32	8.02	6.53	−6.53	−0.92	3.73
Tyr-Cys (z = 0) Tyr/Cys	83.29/83.76	7.82	6.49	−6.49	−0.95	3.72
Tyr-Asn (z = 0)	87.95	8.14	6.99	−6.99	−0.89	3.44
Tyr-Asp(z = −1)	78.98	4.35	2.85	−2.85	1.60	0.63
Tyr-Gln (z = 0)	80.84	8.12	6.49	−6.49	−0.85	3.67
Tyr-Glu (z = −1)	76.68	4.62	2.81	−2.81	1.68	0.57
Tyr-Lys (z = 1)	86.85	10.78	8.40	−8.40	−4.17	6.28
Tyr-Arg(z = 1)	86.70	9.09	8.49	−8.49	−4.26	6.38
Tyr-Ser (z = 0)	79.56	8.21	6.48	−6.48	−0.90	3.69
Tyr-Thr (z = 0)	83.08	7.80	6.26	−6.26	−0.93	3.59
Tyr-His (z = 0) Tyr/His	84.78/82.68	7.32	6.01	−6.01	−1.17	3.59
Tyr-Trp (z = 0) Tyr/Trp	78.34/80.22	6.99	5.43	−5.43	−0.89	3.16
Tyr-Met (z = 0)	83.98	7.42	6.23	−6.23	−0.94	3.58
